# Tris(2-amino-1,3-thia­zolium) hydrogen sulfate sulfate monohydrate

**DOI:** 10.1107/S1600536811046010

**Published:** 2011-11-05

**Authors:** Irena Matulková, Ivan Němec, Jaroslav Cihelka, Michaela Pojarová, Michal Dušek

**Affiliations:** a Department of Inorganic Chemistry, Faculty of Science, Charles University in Prague, Hlavova 2030, 128 40 Prague 2, Czech Republic; b Institute of Physics, AS CR, v.v.i., Na Slovance 2, 182 21 Praha 8, Czech Republic

## Abstract

The centrosymmetric crystal structure of the novel semi-organic compound, 3C_3_H_5_N_2_S^+^·HSO_4_^−^·SO_4_^2−^·H_2_O, is based on chains of alternating anions and water mol­ecules (formed by O—H⋯O hydrogen bonds). The chains are inter­connected with the 2-amino-1,3-thia­zolium cations *via* strong N—H⋯O and weak C—H⋯O hydrogen-bonding inter­actions into a three-dimensional network.

## Related literature

For the use of 2-amino­thia­zole as organo-functionalized films of TiO_2_ or SiO_2_ particles for decontamination of aqueous media or ethanol fuel, see: Cristante *et al.* (2007 ▶); Takeuchi *et al.* (2007 ▶) and for the use of 2-amino­thia­zole and its derivatives as anti­corrosive films, see: Ciftci *et al.* (2011 ▶). For use of 2-amino­thia­zole and its derivatives in medicine, see: De *et al.* (2008 ▶); Aridoss *et al.* (2009 ▶); Franklin *et al.* (2008 ▶); Li *et al.* (2009 ▶); Alexandru *et al.* (2010 ▶). For the non-linear optical properties of similar amino­triazole compounds, see: Yesilel *et al.* (2008 ▶); Matulková *et al.* (2007 ▶, 2008 ▶).
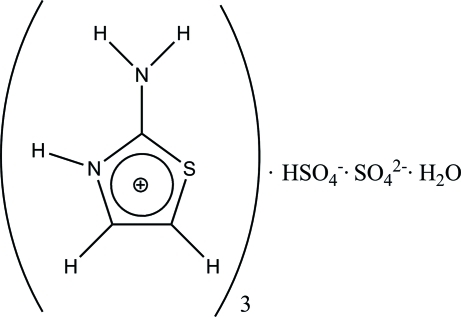


## Experimental

### 

#### Crystal data

3C_3_H_5_N_2_S^+^·HSO_4_^−^·SO_4_^2−^·H_2_O
*M* *_r_* = 514.59
Monoclinic, 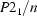

*a* = 11.6418 (1) Å
*b* = 9.8549 (1) Å
*c* = 17.4291 (1) Å
β = 90.3853 (7)°
*V* = 1999.57 (3) Å^3^
*Z* = 4
Cu *K*α radiation
μ = 5.89 mm^−1^
*T* = 120 K
0.52 × 0.15 × 0.10 mm

#### Data collection

Agilent Xcalibur Atlas Gemini ultra diffractometer
Absorption correction: multi-scan (*CrysAlis PRO*; Agilent, 2010 ▶) *T*_min_ = 0.136, *T*_max_ = 1.000
25544 measured reflections
3558 independent reflections
3455 reflections with *I* > 2σ(*I*)
*R*_int_ = 0.040

#### Refinement

*R*[*F* ^2^ > 2σ(*F* ^2^)] = 0.029
*wR*(*F* ^2^) = 0.075
*S* = 1.05
3558 reflections
280 parameters
H atoms treated by a mixture of independent and constrained refinement
Δρ_max_ = 0.33 e Å^−3^
Δρ_min_ = −0.68 e Å^−3^

### 

Data collection: *CrysAlis PRO* (Agilent, 2010 ▶); cell refinement: *CrysAlis PRO*; data reduction: *CrysAlis PRO*; program(s) used to solve structure: *SHELXS97* (Sheldrick, 2008 ▶); program(s) used to refine structure: *SHELXL97* (Sheldrick, 2008 ▶); molecular graphics: *PLATON* (Spek, 2009 ▶); software used to prepare material for publication: *publCIF* (Westrip, 2010 ▶).

## Supplementary Material

Crystal structure: contains datablock(s) I, global. DOI: 10.1107/S1600536811046010/vm2128sup1.cif

Structure factors: contains datablock(s) I. DOI: 10.1107/S1600536811046010/vm2128Isup2.hkl

Supplementary material file. DOI: 10.1107/S1600536811046010/vm2128Isup3.cml

Additional supplementary materials:  crystallographic information; 3D view; checkCIF report

## Figures and Tables

**Table 1:** Hydrogen-bond geometry (Å, °)

*D*—H⋯*A*	*D*—H	H⋯*A*	*D*⋯*A*	*D*—H⋯*A*
O3—H1*O*3⋯O9^i^	0.91 (2)	1.56 (2)	2.474 (2)	178 (2)
O5—H2*O*5⋯O8	0.84 (3)	1.91 (3)	2.7376 (19)	166 (2)
O5—H1*O*5⋯O4	0.75 (3)	2.03 (3)	2.7628 (19)	166 (3)
N1—H1*N*1⋯O2^ii^	0.83 (2)	2.12 (2)	2.904 (2)	158 (2)
N1—H2*N*1⋯O7^iii^	0.83 (3)	2.04 (3)	2.869 (2)	172 (2)
N2—H1*N*2⋯O6^iii^	0.80 (3)	1.89 (3)	2.695 (2)	175 (2)
N3—H1*N*3⋯O1^iii^	0.83 (3)	1.91 (3)	2.730 (2)	179 (3)
N4—H1*N*4⋯O7^ii^	0.80 (3)	2.23 (3)	2.968 (2)	156 (3)
N4—H2*N*4⋯O2^iii^	0.83 (3)	2.14 (3)	2.958 (2)	167 (2)
N5—H1*N*5⋯O8^iv^	0.88 (2)	2.01 (2)	2.870 (2)	165.0 (19)
N5—H2*N*5⋯O5	0.83 (3)	1.94 (3)	2.755 (2)	164 (2)
N6—H1*N*6⋯O6^iv^	0.83 (2)	2.02 (2)	2.814 (2)	160 (2)
C8—H8⋯O4^iii^	0.93	2.44	3.238 (2)	144
C9—H9⋯O5^ii^	0.93	2.53	3.380 (2)	152

Symmetry codes: (i) 
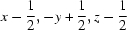
; (ii) 
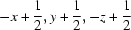
; (iii) 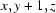; (iv) 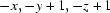.

**Fig. 1.**
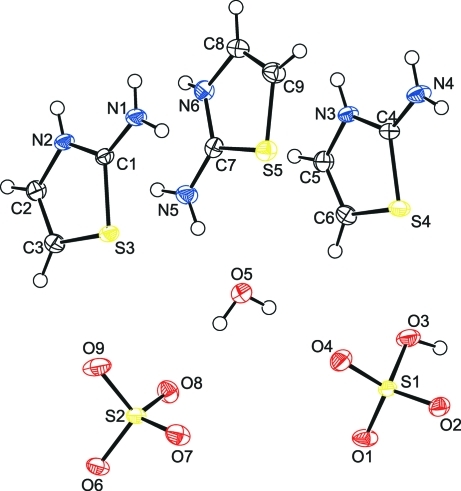
The atom-labelling scheme of tris(2-aminothiazolium) hydrogen sulfate - sulfate monohydrate. Displacement ellipsoids are drawn at the 50% probability level.

**Fig. 2.**
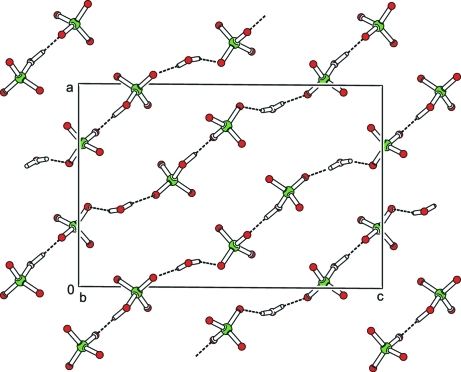
A packing scheme of the anions and water molecules in the crystals of tris(2-aminothiazolium) hydrogen sulfate - sulfate monohydrate (projection along [010]). The dashed lines indicates the hydrogen bonds.

**Fig. 3.**
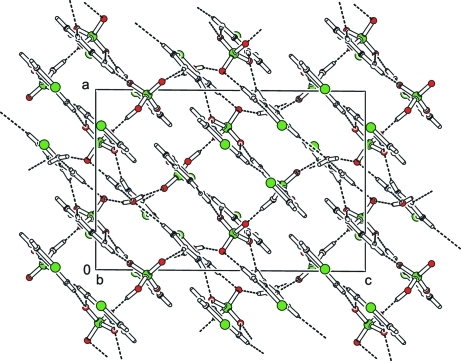
A packing scheme of the structure of tris(2-aminothiazolium) hydrogen sulfate - sulfate monohydrate (projection along [010]). Hydrogen bonds are indicated by dashed lines.

## supplementary crystallographic information

### Comment

Recent ecological studies show interest in TiO_2_ or SiO_2_ particles modified by 2-aminothiazole. Sorption and photocatalytic reduction or degradation in aqueous solutions by 2-aminothiazole modified TiO_2_ particles has been described (Cristante *et al.*, 2007). Metal impurities in ethanol fuel can be detected by the electrodes modified with 2-aminothiazole organo-functionalized silica (Takeuchi *et al.*, 2007).

The last few years, there is a huge interest in the field of fundamental research of conducting polymers such as polyaminothiazoles (Ciftci *et al.*, 2011).

Natural and synthetic thiazole derivatives find applications as antioxidants, antibacterial drugs and fungicide (De *et al.* 2008; Aridoss *et al.*, 2009). Anti-inflammatory, analgesic and antipyretic activities were observed for thiazolyl and benzothiazolyl derivatives (Franklin *et al.*, 2008). The medical application of metal complexes of 2-aminothiazole and its derivatives involve their use as inhibitors of human cancer, Alzheimers disease, antitumor activity and activity against leukemia (Li *et al.*, 2009; Alexandru *et al.*, 2010).

The salt, bis(2-aminothiazolium) squarate dihydrate (Yesilel *et al.*, 2008), was widely studied for hydrogen bond interactions, which are very attractive in the biological activities, biochemical processes, material and supramolecular chemistry.

The preparation of the title compound was motivated by the previous study on salts or cocrystals of similar aminotriazoles (Matulková *et al.*, 2007, 2008). 2-aminothiazole compounds easily build hydrogen bonding networks, very useful for the preparation of materials with potential non-linear optical properties. Unfortunately, the title compound crystallizes in the centrosymmetric space group *P*2_1_/n, which excludes the second order non-linear optical properties.

The crystal structure of the title compound (Fig. 1) is based on chains of alternating anions and water molecules formed *via* O—H···O hydrogen bonds with donor-acceptor distances in the interval 2.474 (2)–2.7628 (19) Å (Fig. 2). The chains are interconnected with 2-aminothiazolium (1+) cations *via* strong N—H···O (2.695 (2)–2.968 (2) Å) and weak C—H···O (3.238 (2)–3.380 (2) Å) hydrogen interactions (Table 1) into a three-dimensional network (Fig. 3). The cation rings are oriented along the axis *b* and are perpendicular to the *ac* plane.

### Experimental

Crystals of the title compound, were obtained from a solution of 1.0 g of 2-aminothiazole (97%, Aldrich) and 0.56 ml of sulfuric acid (96%, Lachema) in 200 ml of water. The solution was left to crystallize at room temperature for several weeks. The colourless crystals obtained were filtered off, washed with methanol and dried in a vacuum desiccator over KOH.

The infrared spectrum was recorded at room temperature using DRIFTS and the nujol or fluorolube mull techniques on a Nicolet Magna 6700 FTIR spectrometer with 2 cm^-1^ resolution and Happ-Genzel apodization in the 400–4000 cm^-1^ region.

FTIR spectrum (cm^-1^): 3315 *s*; 3235 *s*; 3119 *s*; 3085 *s*; 2980 *m*; 2930 *m*; 2850 *m*; 2758 *m*; 1727 *w*; 1621 *s*; 1576 *m*; 1434 *m*; 1398 *w*; 1339 *w*; 1276 *m*; 1189 *mb*; 1079 *m*; 1062 *m*; 1028 *m*; 1005 *mb*; 887 *mb*; 868 *m*; 860 *m*; 772 *mb*; 739 *sh*; 732 *m*; 698 *m*; 639 *m*; 600 *s*; 592 *sh*; 562 *s*; 550 *sh*; 494 *m*; 408 *mb*.

The Raman spectrum of polycrystalline sample was recorded at room temperature on a Thermo Scientific DXR Raman microscope interface to on Olympus microscope (3 cm^-1^ resolution, 780 nm diode laser excitation, 15–20 mW power at the sample) in the 50–3300 cm^-1^ region.

Raman spectrum (cm^-1^): 3164 *m*; 3157 *m*; 3085 *m*; 3082 *m*; 3074 *w*; 1684 *w*; 1606 *w*; 1557 *m*; 1410 *w*; 1366 *m*; 1282 *m*; 1174 *m*; 1076 *mb*; 1032 *sh*; 977 *m*; 901 *wb*; 879 *wb*; 863 *wb*; 748 *vs*; 708 *m*; 593 *w*; 568 *m*; 418 *w*; 395 *m*; 267 *vw*; 113 *sh*; 89 *m*; 79 *m*; 62 *m*.

### Refinement

H atoms attached to C and N atoms were calculated in geometrically idealized positions, C*sp*^2^ - H = 0.93 Å, and constrained to ride on their parent atoms, with *U*_iso_(H) = 1.2 *U*_eq_(C). The positions of H atoms attached to O and N atoms were localized in difference Fourier maps, the distances were left unrestrained and the hydrogen atom were refined isotropically with *U*_iso_(H) = 1.2 *U*_eq_ of parent atom.

### Crystal data

**Table:**

3C_3_H_5_N_2_S^+^·HSO_4_^−^·SO_4_^2^^−^·H_2_O	*F*(000) = 1064
*M**_r_* = 514.59	*D*_x_ = 1.709 Mg m^−^^3^
Monoclinic, *P*2_1_/*n*	Cu *K*α radiation, λ = 1.54178 Å
Hall symbol: -P 2yn	Cell parameters from 19931 reflections
*a* = 11.6418 (1) Å	θ = 3.8–66.9°
*b* = 9.8549 (1) Å	µ = 5.89 mm^−^^1^
*c* = 17.4291 (1) Å	*T* = 120 K
β = 90.3853 (7)°	Plate, colourless
*V* = 1999.57 (3) Å^3^	0.52 × 0.15 × 0.10 mm
*Z* = 4	

### Data collection

**Table:**

Agilent Xcalibur Atlas Gemini ultra diffractometer	3558 independent reflections
Radiation source: Enhance Ultra (Cu) X-ray Source	3455 reflections with *I* > 2σ(*I*)
mirror	*R*_int_ = 0.040
Detector resolution: 10.3784 pixels mm^-1^	θ_max_ = 67.0°, θ_min_ = 4.6°
Rotation method data acquisition using ω scans	*h* = −13→13
Absorption correction: multi-scan (*CrysAlis PRO*; Agilent, 2010)	*k* = −11→11
*T*_min_ = 0.136, *T*_max_ = 1.000	*l* = −18→20
25544 measured reflections	

### Refinement

**Table:**

Refinement on *F*^2^	Primary atom site location: structure-invariant direct methods
Least-squares matrix: full	Secondary atom site location: difference Fourier map
*R*[*F*^2^ > 2σ(*F*^2^)] = 0.029	Hydrogen site location: inferred from neighbouring sites
*wR*(*F*^2^) = 0.075	H atoms treated by a mixture of independent and constrained refinement
*S* = 1.05	*w* = 1/[σ^2^(*F*_o_^2^) + (0.0412*P*)^2^ + 1.5439*P*] where *P* = (*F*_o_^2^ + 2*F*_c_^2^)/3
3558 reflections	(Δ/σ)_max_ = 0.001
280 parameters	Δρ_max_ = 0.33 e Å^−^^3^
0 restraints	Δρ_min_ = −0.68 e Å^−^^3^

### Special details

**Table:**

Geometry. All e.s.d.'s (except the e.s.d. in the dihedral angle between two l.s. planes) are estimated using the full covariance matrix. The cell e.s.d.'s are taken into account individually in the estimation of e.s.d.'s in distances, angles and torsion angles; correlations between e.s.d.'s in cell parameters are only used when they are defined by crystal symmetry. An approximate (isotropic) treatment of cell e.s.d.'s is used for estimating e.s.d.'s involving l.s. planes.
Refinement. Refinement of *F*^2^ against ALL reflections. The weighted *R*-factor *wR* and goodness of fit *S* are based on *F*^2^, conventional *R*-factors *R* are based on *F*, with *F* set to zero for negative *F*^2^. The threshold expression of *F*^2^ > σ(*F*^2^) is used only for calculating *R*-factors(gt) *etc*. and is not relevant to the choice of reflections for refinement. *R*-factors based on *F*^2^ are statistically about twice as large as those based on *F*, and *R*- factors based on ALL data will be even larger. The hydrogen atoms were localized from the difference Fourier map. Despite of that,all hydrogen atoms connected to C were constrained to ideal positions. The N—H and O—H distances were left unrestrained. The isotropic temperature parameters of hydrogen atoms were calculated as 1.2**U*_eq_ of the parent atom.

### Fractional atomic coordinates and isotropic or equivalent isotropic displacement parameters (Å^2^)

**Table:**

	*x*	*y*	*z*	*U*_iso_*/*U*_eq_	
C1	0.29359 (14)	0.74559 (18)	0.51444 (9)	0.0168 (3)	
C2	0.16124 (15)	0.69841 (19)	0.60694 (10)	0.0203 (4)	
H2	0.1057	0.7191	0.6432	0.024*	
C3	0.19494 (15)	0.57200 (18)	0.59119 (10)	0.0207 (4)	
H3	0.1662	0.4948	0.6150	0.025*	
O1	−0.10315 (11)	0.07767 (13)	0.22736 (8)	0.0248 (3)	
O2	0.03550 (11)	0.11665 (13)	0.12781 (7)	0.0231 (3)	
O3	−0.10426 (12)	0.29105 (13)	0.15778 (8)	0.0261 (3)	
H1O3	−0.153 (2)	0.265 (2)	0.1193 (14)	0.031*	
O4	0.04573 (11)	0.24617 (13)	0.24574 (7)	0.0264 (3)	
O5	0.11893 (12)	0.41726 (14)	0.36173 (8)	0.0243 (3)	
H2O5	0.127 (2)	0.354 (3)	0.3937 (14)	0.029*	
H1O5	0.091 (2)	0.380 (3)	0.3292 (15)	0.029*	
O6	0.16351 (11)	0.06136 (12)	0.56939 (7)	0.0232 (3)	
O7	0.29107 (11)	0.10060 (13)	0.46267 (8)	0.0259 (3)	
O8	0.10714 (11)	0.21584 (13)	0.46956 (7)	0.0239 (3)	
O9	0.25934 (12)	0.27724 (13)	0.55539 (8)	0.0308 (3)	
S1	−0.02774 (3)	0.17660 (4)	0.19126 (2)	0.01635 (10)	
S2	0.20561 (3)	0.16181 (4)	0.51308 (2)	0.01708 (10)	
S3	0.29952 (4)	0.57034 (4)	0.52038 (2)	0.01888 (10)	
N1	0.35685 (14)	0.82017 (17)	0.46841 (9)	0.0221 (3)	
H1N1	0.399 (2)	0.780 (2)	0.4378 (14)	0.026*	
H2N1	0.344 (2)	0.903 (3)	0.4650 (13)	0.026*	
N2	0.21729 (13)	0.79555 (16)	0.56378 (8)	0.0178 (3)	
H1N2	0.2031 (19)	0.875 (3)	0.5679 (12)	0.021*	
C7	0.08543 (14)	0.75482 (19)	0.37523 (10)	0.0180 (3)	
C8	0.12455 (15)	0.96823 (19)	0.33003 (10)	0.0223 (4)	
H8	0.1162	1.0619	0.3267	0.027*	
C9	0.19866 (16)	0.89764 (19)	0.28789 (11)	0.0243 (4)	
H9	0.2476	0.9356	0.2518	0.029*	
S5	0.19216 (4)	0.72483 (5)	0.30915 (2)	0.02143 (10)	
N5	0.03629 (14)	0.65995 (17)	0.41708 (9)	0.0223 (3)	
H1N5	−0.012 (2)	0.683 (2)	0.4538 (14)	0.027*	
H2N5	0.055 (2)	0.580 (3)	0.4078 (13)	0.027*	
N6	0.06105 (13)	0.88732 (16)	0.37947 (8)	0.0200 (3)	
H1N6	0.003 (2)	0.915 (2)	0.4024 (13)	0.024*	
S4	−0.01139 (4)	0.57716 (4)	0.14954 (2)	0.020	
C4	0.00150 (14)	0.75220 (18)	0.15074 (10)	0.017	
C5	−0.13895 (15)	0.71377 (19)	0.23921 (10)	0.022	
H5	−0.1933	0.7379	0.2757	0.026*	
C6	−0.11839 (16)	0.58563 (19)	0.21834 (10)	0.0230 (4)	
H6	−0.1565	0.5106	0.2381	0.028*	
N3	−0.07096 (12)	0.80736 (16)	0.20091 (8)	0.0176 (3)	
H1N3	−0.0797 (19)	0.889 (3)	0.2095 (12)	0.021*	
N4	0.07243 (14)	0.82190 (17)	0.10758 (10)	0.0238 (3)	
H1N4	0.116 (2)	0.782 (3)	0.0809 (14)	0.029*	
H2N4	0.073 (2)	0.906 (3)	0.1111 (13)	0.029*	

### Atomic displacement parameters (Å^2^)

**Table:**

	*U*^11^	*U*^22^	*U*^33^	*U*^12^	*U*^13^	*U*^23^
C1	0.0194 (8)	0.0133 (8)	0.0176 (8)	0.0016 (6)	−0.0015 (6)	−0.0017 (6)
C2	0.0194 (8)	0.0210 (9)	0.0206 (8)	−0.0009 (7)	0.0042 (7)	−0.0008 (7)
C3	0.0222 (8)	0.0190 (9)	0.0208 (8)	−0.0029 (7)	0.0025 (7)	0.0020 (7)
O1	0.0255 (6)	0.0157 (6)	0.0333 (7)	−0.0002 (5)	0.0138 (5)	0.0015 (5)
O2	0.0280 (7)	0.0184 (6)	0.0231 (6)	−0.0021 (5)	0.0101 (5)	−0.0029 (5)
O3	0.0332 (7)	0.0145 (6)	0.0305 (7)	0.0028 (5)	−0.0066 (6)	−0.0013 (5)
O4	0.0310 (7)	0.0227 (7)	0.0255 (7)	−0.0009 (6)	−0.0037 (5)	−0.0041 (5)
O5	0.0306 (7)	0.0198 (7)	0.0225 (7)	−0.0021 (5)	0.0012 (6)	−0.0019 (6)
O6	0.0280 (6)	0.0156 (6)	0.0260 (6)	0.0041 (5)	0.0109 (5)	0.0048 (5)
O7	0.0255 (7)	0.0224 (7)	0.0298 (7)	0.0039 (5)	0.0122 (5)	0.0043 (6)
O8	0.0229 (6)	0.0261 (7)	0.0228 (6)	0.0032 (5)	0.0004 (5)	0.0026 (5)
O9	0.0349 (7)	0.0148 (7)	0.0425 (8)	0.0003 (5)	−0.0122 (6)	−0.0016 (6)
S1	0.0188 (2)	0.0124 (2)	0.0179 (2)	−0.00083 (15)	0.00330 (16)	−0.00037 (15)
S2	0.0185 (2)	0.0126 (2)	0.0201 (2)	0.00032 (15)	0.00292 (16)	0.00103 (15)
S3	0.0230 (2)	0.0126 (2)	0.0210 (2)	0.00234 (15)	0.00299 (16)	−0.00110 (15)
N1	0.0297 (8)	0.0143 (8)	0.0223 (8)	0.0016 (6)	0.0099 (6)	0.0001 (6)
N2	0.0203 (7)	0.0123 (7)	0.0208 (7)	0.0027 (6)	0.0033 (6)	−0.0020 (6)
C7	0.0157 (8)	0.0204 (9)	0.0178 (8)	0.0019 (7)	−0.0013 (6)	−0.0020 (7)
C8	0.0240 (9)	0.0175 (9)	0.0253 (9)	−0.0007 (7)	−0.0001 (7)	0.0022 (7)
C9	0.0233 (9)	0.0227 (10)	0.0269 (9)	−0.0025 (7)	0.0038 (7)	0.0029 (8)
S5	0.0196 (2)	0.0205 (2)	0.0243 (2)	0.00299 (16)	0.00520 (17)	−0.00179 (17)
N5	0.0250 (8)	0.0178 (8)	0.0243 (8)	0.0031 (6)	0.0056 (6)	0.0015 (6)
N6	0.0188 (7)	0.0208 (8)	0.0204 (7)	0.0041 (6)	0.0044 (6)	−0.0007 (6)
S4	0.025	0.013	0.024	0.000	0.004	0.002
C4	0.018	0.013	0.021	0.000	0.000	0.001
C5	0.022	0.022	0.021	0.001	0.006	−0.001
C6	0.0271 (9)	0.0211 (9)	0.0210 (9)	−0.0051 (7)	0.0040 (7)	0.0028 (7)
N3	0.0189 (7)	0.0120 (7)	0.0220 (7)	0.0011 (6)	0.0032 (6)	−0.0014 (6)
N4	0.0251 (8)	0.0148 (8)	0.0317 (9)	−0.0014 (6)	0.0128 (7)	−0.0032 (7)

### Geometric parameters (Å, °)

**Table:**

C1—N1	1.317 (2)	C7—N5	1.319 (2)
C1—N2	1.335 (2)	C7—N6	1.338 (2)
C1—S3	1.7315 (18)	C7—S5	1.7254 (17)
C2—C3	1.335 (3)	C8—C9	1.333 (3)
C2—N2	1.384 (2)	C8—N6	1.390 (2)
C2—H2	0.9300	C8—H8	0.9300
C3—S3	1.7395 (18)	C9—S5	1.7446 (19)
C3—H3	0.9300	C9—H9	0.9300
O1—S1	1.4576 (13)	N5—H1N5	0.88 (2)
O2—S1	1.4577 (12)	N5—H2N5	0.83 (3)
O3—S1	1.5490 (13)	N6—H1N6	0.83 (2)
O3—H1O3	0.91 (3)	S4—C4	1.7316 (18)
O4—S1	1.4465 (13)	S4—C6	1.7369 (18)
O5—H2O5	0.84 (3)	C4—N4	1.314 (2)
O5—H1O5	0.75 (3)	C4—N3	1.335 (2)
O6—S2	1.4797 (13)	C5—C6	1.336 (3)
O7—S2	1.4620 (13)	C5—N3	1.389 (2)
O8—S2	1.4701 (13)	C5—H5	0.9300
O9—S2	1.4908 (14)	C6—H6	0.9300
N1—H1N1	0.83 (3)	N3—H1N3	0.83 (2)
N1—H2N1	0.83 (3)	N4—H1N4	0.80 (3)
N2—H1N2	0.81 (2)	N4—H2N4	0.83 (3)
			
N1—C1—N2	124.34 (17)	N5—C7—S5	124.39 (14)
N1—C1—S3	124.81 (14)	N6—C7—S5	110.97 (13)
N2—C1—S3	110.84 (13)	C9—C8—N6	113.02 (17)
C3—C2—N2	113.17 (16)	C9—C8—H8	123.5
C3—C2—H2	123.4	N6—C8—H8	123.5
N2—C2—H2	123.4	C8—C9—S5	111.31 (14)
C2—C3—S3	111.26 (13)	C8—C9—H9	124.3
C2—C3—H3	124.4	S5—C9—H9	124.3
S3—C3—H3	124.4	C7—S5—C9	90.40 (9)
S1—O3—H1O3	115.0 (15)	C7—N5—H1N5	119.7 (15)
H2O5—O5—H1O5	101 (3)	C7—N5—H2N5	116.7 (16)
O4—S1—O1	112.89 (8)	H1N5—N5—H2N5	124 (2)
O4—S1—O2	113.00 (8)	C7—N6—C8	114.30 (15)
O1—S1—O2	111.42 (7)	C7—N6—H1N6	121.2 (16)
O4—S1—O3	103.77 (8)	C8—N6—H1N6	123.1 (15)
O1—S1—O3	107.65 (8)	C4—S4—C6	90.36 (9)
O2—S1—O3	107.55 (8)	N4—C4—N3	124.35 (17)
O7—S2—O8	111.76 (8)	N4—C4—S4	124.64 (14)
O7—S2—O6	110.62 (7)	N3—C4—S4	111.01 (13)
O8—S2—O6	108.90 (8)	C6—C5—N3	113.14 (16)
O7—S2—O9	109.09 (8)	C6—C5—H5	123.4
O8—S2—O9	107.58 (8)	N3—C5—H5	123.4
O6—S2—O9	108.80 (8)	C5—C6—S4	111.34 (14)
C1—S3—C3	90.30 (8)	C5—C6—H6	124.3
C1—N1—H1N1	117.7 (16)	S4—C6—H6	124.3
C1—N1—H2N1	119.3 (16)	C4—N3—C5	114.15 (15)
H1N1—N1—H2N1	122 (2)	C4—N3—H1N3	126.5 (15)
C1—N2—C2	114.42 (15)	C5—N3—H1N3	119.3 (15)
C1—N2—H1N2	123.7 (16)	C4—N4—H1N4	118.6 (18)
C2—N2—H1N2	121.9 (15)	C4—N4—H2N4	118.9 (16)
N5—C7—N6	124.62 (16)	H1N4—N4—H2N4	122 (2)

### Hydrogen-bond geometry (Å, °)

**Table:**

*D*—H···*A*	*D*—H	H···*A*	*D*···*A*	*D*—H···*A*
O3—H1O3···O9^i^	0.91 (2)	1.56 (2)	2.474 (2)	178 (2)
O5—H2O5···O8	0.84 (3)	1.91 (3)	2.7376 (19)	166 (2)
O5—H1O5···O4	0.75 (3)	2.03 (3)	2.7628 (19)	166 (3)
N1—H1N1···O2^ii^	0.83 (2)	2.12 (2)	2.904 (2)	158 (2)
N1—H2N1···O7^iii^	0.83 (3)	2.04 (3)	2.869 (2)	172 (2)
N2—H1N2···O6^iii^	0.80 (3)	1.89 (3)	2.695 (2)	175 (2)
N3—H1N3···O1^iii^	0.83 (3)	1.91 (3)	2.730 (2)	179 (3)
N4—H1N4···O7^ii^	0.80 (3)	2.23 (3)	2.968 (2)	156 (3)
N4—H2N4···O2^iii^	0.83 (3)	2.14 (3)	2.958 (2)	167 (2)
N5—H1N5···O8^iv^	0.88 (2)	2.01 (2)	2.870 (2)	165.0 (19)
N5—H2N5···O5	0.83 (3)	1.94 (3)	2.755 (2)	164 (2)
N6—H1N6···O6^iv^	0.83 (2)	2.02 (2)	2.814 (2)	160 (2)
C8—H8···O4^iii^	0.93	2.44	3.238 (2)	144
C9—H9···O5^ii^	0.93	2.53	3.380 (2)	152

Symmetry codes: (i) *x*−1/2, −*y*+1/2, *z*−1/2; (ii) −*x*+1/2, *y*+1/2, −*z*+1/2; (iii) *x*, *y*+1, *z*; (iv) −*x*, −*y*+1, −*z*+1.

## References

[bb1] Agilent (2010). *CrysAlis PRO* Agilent Technologies, Yarnton, England.

[bb2] Alexandru, M.-G., Velikovic, T. C., Jitaru, I., Grguric-Sipka, S. & Draghici, C. (2010). *Cent. Eur. J. Chem.* **8**, 639–645.

[bb3] Aridoss, G., Amirthaganesan, S., Kim, M. S., Kim, J. T. & Jeong, Y. T. (2009). *Eur. J. Med. Chem.* **44**, 4199–4210. 10.1016/j.ejmech.2009.05.015 19535178

[bb4] Ciftci, H., Testereci, H. N. & Oktem, Z. (2011). *Polym. Bull.* **66**, 747–760.

[bb5] Cristante, V. M., Jorge, S. M. A., Valente, J. P. S., Saeki, M. J., Florentino, A. O. & Padilha, P. M. (2007). *Thin Solid Films*, **515**, 5334–5340.

[bb6] De, S., Adhikari, S., Tilak-Jain, J., Menon, V. P. & Devasagayam, T. P. A. (2008). *Chem. Biol. Interact.* **173**, 215–223. 10.1016/j.cbi.2008.03.011 18466888

[bb7] Franklin, P. X., Pillai, A. D., Rathod, P. D., Yerande, S., Nivsarkar, M., Padh, H., Vasu, K. K. & Sudarsanam, V. (2008). *Eur. J. Med. Chem.* **43**, 129–134. 10.1016/j.ejmech.2007.02.008 17467123

[bb8] Li, J., Du, J., Xia, L., Liu, H., Yao, X. & Liu, M. (2009). *Anal. Chim. Acta*, **631**, 29–39. 10.1016/j.aca.2008.10.026 19046675

[bb9] Matulková, I., Němec, I., Císařová, I., Němec, P. & Mička, Z. (2007). *J. Mol. Struct.* **834–836**, 328–335.

[bb10] Matulková, I., Němec, I., Teubner, K., Němec, P. & Mička, Z. (2008). *J. Mol. Struct.* **837**, 46–60.

[bb11] Sheldrick, G. M. (2008). *Acta Cryst.* A**64**, 112–122. 10.1107/S0108767307043930 18156677

[bb12] Spek, A. L. (2009). *Acta Cryst.* D**65**, 148–155. 10.1107/S090744490804362X PMC2631630 19171970

[bb13] Takeuchi, R. M., Santos, A. L., Padilha, P. M. & Stradiotto, N. R. (2007). *Talanta*, **71**, 771–777. 10.1016/j.talanta.2006.05.035 19071372

[bb14] Westrip, S. P. (2010). *J. Appl. Cryst.* **43**, 920–925.

[bb15] Yesilel, O. Y., Odabasoglu, M. & Buyukgungor, O. (2008). *J. Mol. Struct.* **874**, 151–158.

